# Risk translation in a compressed meritocracy: a sociological and social-psychological analysis of the Zhang Xuefeng phenomenon

**DOI:** 10.3389/fsoc.2026.1871640

**Published:** 2026-08-13

**Authors:** Hugh Jiliang Liu

**Affiliations:** 1 International Business Unit, Business and Law Cluster, Hanze University of Applied Sciences, Groningen, Netherlands; 2 Department of Psychology, Faculty of Behavioral and Social Sciences, University of Groningen, Groningen, Netherlands

**Keywords:** China, credentialism, educational inequality, gaokao, risk society, social mobility, social psychology

## Abstract

Zhang Xuefeng (1984–2026) was a prominent Chinese education entrepreneur, lecturer, social-media influencer, and education consultant known for offering blunt, employment-oriented advice on university choice, major selection, and career planning. His public influence made visible a broader social problem: how families make educational decisions when formal schooling is viewed as the primary route to upward mobility, while the labor market appears increasingly uncertain and consequential. This article regards the “Zhang Xuefeng phenomenon” as a revealing case of how educational authority, social inequality, and family decision-making are reorganized under intensified competition. It argues that the phenomenon can be understood as risk translation within a compressed meritocracy: the conversion of broad social uncertainties into concrete decision rules within an education-to-work system in which a few high-stakes transitions appear to determine long-term security. From a sociological perspective, the phenomenon reflects the growing tendency to treat education not only as self-development but also as a household risk-management strategy in a compressed meritocracy. From a social-psychological perspective, it reflects how uncertainty, scarcity, social comparison, and the search for credible authority may make pragmatic advice emotionally compelling, especially for students and families with fewer socioeconomic resources or limited economic buffers. For educators, the implication is to develop more democratic guidance systems that combine labor-market transparency, equity-sensitive advising, developmental purpose, mental-health protection, and attention to both employment realities and students' broader developmental possibilities.

## 1 Introduction

Zhang Xuefeng (1984–2026) was a Chinese education entrepreneur, lecturer, and social media influencer whose public identity was built around college entrance and graduate school counseling. He was the founder and chief executive of Suzhou Fengxue Weilai Education Technology, and the most visible figure in China's education-consulting industry (Zou, 2026). Zhang gained national attention after a viral 2016 video in which he explained complex information about elite Chinese universities in a fast-paced, humorous, 7 min presentation (Quan, 2023). By 2026, he had accumulated more than 40 million online followers across platforms (South China Morning Post, 2026). He had a personal brand closely associated with practical guidance for anxious families navigating the *Gaokao* (i.e., China's national college entrance examination) and postgraduate entrance systems (Jiang, 2024).

Zhang's death following a sudden cardiac arrest on March 24, 2026, at age 41, produced a striking scene of collective mourning. Although no official attendance figure appears to have been released, reports described thousands of people lining up outside the Suzhou Funeral Home to bid farewell, and one unofficial account estimated, without official confirmation, that the number of mourners who arrived spontaneously from across China may have exceeded 10,000 (Liu J. et al., 2026). This unusually large public turnout for an educational consultant suggests that Zhang had become more than an online celebrity. For many families, he might have symbolized a rare source of practical guidance in an education system experienced as uncertain, competitive, and consequential for social mobility. The funeral thus might have become not only an expression of grief for an individual, but also a public sign of the emotional dependency, trust, and social anxiety that had accumulated around his role in Chinese educational life. Zhang's death turned an already influential figure into a symbolic object of public grief and reflection (Global Times, 2026; Zou, 2026).

The *Zhang Xuefeng phenomenon* refers to the widespread public attention, trust, and controversy generated by Zhang's employment-oriented educational advice, particularly his influence on how Chinese students and families understand university choice, major selection, and career planning within China's highly competitive education system. The most controversial aspect of Zhang's advice was not the claim that education matters, but the implication that education matters too much to be approached romantically by families without substantial economic protection. He often framed major selection as an unequal-risk decision. Privileged students could afford exploration, delayed returns, and the pursuit of interests, students from less advantaged socioeconomic backgrounds had to prioritize employability, occupational closure, civil-service opportunities, and predictable income. His message was therefore heard by many as a class-coded warning: “Do not confuse elite ideals with mass survival”.

For educators, this phenomenon is significant because it exposes a mismatch between the moral language of schooling and the lived calculations of students and parents. Educational institutions frequently invoke ideals such as self-realization, holistic development, civic formation, and the cultivation of interest. Families under mobility pressure often experience education as triage, choosing the path that minimizes regret, avoids unemployment, protects parents' investment, and keeps open the possibility of a stable adult life. The Zhang Xuefeng phenomenon dramatizes this mismatch. It reflects that when institutional guidance is perceived as abstract, underinformed, or class-blind, families may turn to charismatic “outsiders” who appear to translate the real rules of the game.

The central argument of this article is that the Zhang Xuefeng phenomenon is best understood as risk translation within a compressed meritocracy. Risk translation refers to the conversion of broad, impersonal, and uncertain social pressures into concrete decision rules that individuals and families can use. This concept draws on social-psychological research on heuristics, uncertainty reduction, and loss aversion under high-stakes conditions (Kahneman and Tversky, 1979, 2013; Kruglanski and Webster, 1996; Tversky and Kahneman, 1974, 1991). Compressed meritocracy refers to an education-to-work system in which opportunity is formally organized around merit, while future security appears to depend on a narrow sequence of high-stakes transitions, including the *Gaokao*, university admission, major selection, and first-job placement. This concept builds on scholarship on positional competition, educational reproduction, and China's exam-centered education system (Brown, 2000; Jia et al., 2025; Mok and Wu, 2016). Within such a context, Zhang's advice may have resonated because it appeared to reduce interpretive ambiguity, translate complex labor-market signals into simplified decision criteria, and offer families a sense of control over choices perceived as consequential for employment security and class mobility (Hang and Zhou, 2025; Li et al., 2024; Ma et al., 2026). The aim of this article is therefore neither to praise nor condemn Zhang, but to explain why his advice may have become socially and psychologically compelling and what educators can learn from that resonance. Accordingly, the article should be read as a theory-generating perspective rather than as an empirical test of audience motives, causal effects, or the accuracy of Zhang's specific recommendations.

## 2 Conceptual frame: educational choice as risk translation in a compressed meritocracy

A useful analysis requires moving beyond the common but limited view that Zhang simply represented pragmatism against idealism. Although this contrast is descriptively appealing, it oversimplifies educational choice by reducing it to personal values, as if some students are idealistic while others are practical. A more appropriate explanation begins with the structure of risk. In Beck (1992) account of risk society, modern institutions generate uncertainties that individuals and families are increasingly expected to manage themselves. Education is a central site of this burden. Structural pressures such as unemployment, industrial change, regional inequality, housing costs, and technological displacement (Liu et al., 2026) are often condensed into one urgent family question: “Which major should my child choose?” Zhang's advice may have become influential in part because it translated these abstract anxieties into concrete and practical choices.

Sociology of education helps explain why Zhang's distinction between “safe” and “risky” majors resonated so strongly. From a human-capital perspective, education is an investment expected to increase future earnings (Becker, 1964). However, credentialist theory suggests that degrees also function as signals, filters, and status markers in the labor market (Collins, 1979; Dore, 1976). As higher education expands, the value of any single credential may decline, intensifying anxiety over the “right” school, major, or pathway. Bourdieu's theory of capital (Bourdieu, 1986, 2018) further clarifies why families are unequally positioned within this competition. In this context, economic capital refers to financial resources that can buffer educational risk; cultural capital refers to familiarity with institutional expectations, academic norms, and valued forms of knowledge; and social capital refers to networks that provide information, guidance, and access to opportunities. Families with more of these resources are often better able to interpret educational signals, consult knowledgeable advisers, secure internships or supplementary credentials, and recover from less successful choices. By contrast, families with fewer financial resources, less familiarity with higher-education and labor-market institutions, and weaker access to career-relevant networks may experience the same educational choices as more consequential and less reversible. Consequently, even expanded educational access may reproduce inequality through subtler advantages, including knowing which choices matter, when to act, whom to consult, and how much educational and occupational risk one can reasonably afford. Zhang's advice became influential in this context because it appeared to offer families with fewer socioeconomic resources a simplified and practical map of an educational market that many experienced as unequal, opaque, and difficult to navigate.

China's recent educational context makes this tension especially visible. Higher education has reached a universal or near-universal stage. The gross enrollment rate has reached 60.8% and 55 million students graduated from colleges and universities during the 2021–2025 period (Xinhua, 2025a). In 2025, 13.35 million candidates registered for the *Gaokao*, slightly below the 2024 record of 13.42 million (Xinhua, 2025a). The Ministry of Education further projected 12.7 million college graduates in 2026, an increase of 480,000 from 2025 (Xinhua, 2025b). At the same time, youth employment remained strained, with the urban unemployment rate for 16–24-year-olds, excluding students, rising to 16.9% in March 2026 and the rate for 25–29-year-olds reaching 7.7% (Reuters, 2026). These figures do not mechanically explain Zhang's influence, but they define the social context in which his advice could plausibly be perceived as credible and compelling.

The concept of compressed meritocracy captures the coexistence of mass aspiration and scarce security. Meritocratic systems promise that effort and examination performance can be converted into upward mobility. Compression occurs when this promise is concentrated into a narrow set of socially legitimate routes perceived as economically secure or defensible, including elite university admission, postgraduate credentials, civil-service examinations, employment in state-owned enterprises, high-technology sectors, and majors perceived as offering relatively stable occupational returns. In Zhang's discourse, “safe” majors were often understood as the fields believed to have clearer pathways to employment, professional certification, or public-sector recruitment, such as computer science, electronic information engineering, clinical medicine, electrical engineering, law, teacher education, accounting, and other applied or professionally oriented programs. Under such conditions, families with fewer socioeconomic resources may be less willing to treat education as open-ended exploration. Instead, they often seek pathways whose risks and returns appear more legible in advance. One plausible source of Zhang's appeal was that he offered precisely this form of legibility. His advice did not merely provide information; it presented a practical map of which educational investments seemed most likely to produce occupational security.

This framework also explains why Zhang's advice was strongly shaped by class position. For affluent families, a degree in the humanities or in a less directly vocational field can often be supplemented by internships, overseas study, postgraduate credentials, social networks, and family resources. For families with limited economic and social buffers, however, the same choice may appear far more consequential. Zhang, therefore, tended to frame major selection less as an expression of personal interest than as a strategy for avoiding downward mobility. This logic is consistent with prospect theory, which argues that potential losses often weigh more heavily than equivalent gains (Kahneman and Tversky, 1979, 2013). Research on scarcity similarly shows that constrained resources narrow attention and heighten sensitivity to urgent trade-offs (Shah et al., 2012). Zhang's advice resonated with this psychology of narrow margins by shifting the central question from “What is the best life in the abstract?” to “Which choice minimizes irreversible loss for those who cannot afford a wrong turn?”

The boundary conditions of these concepts are also important. Risk translation and compressed meritocracy are most applicable in contexts where educational choices are perceived as high stakes, closely linked to employment outcomes, and made under conditions of unequal information and limited institutional guidance. The concepts may therefore be relevant beyond the Zhang Xuefeng case, including to other Chinese edu-influencers, postgraduate-admission advisers, civil-service examination coaches, and international contexts in which credential inflation, positional competition, and uncertain school-to-work transitions heighten the perceived consequences of educational choice (Hang and Zhou, 2025; Ma et al., 2026). However, their applicability is more limited in educational systems where vocational, academic, and work-based pathways are more diverse, reversible, and institutionally supported (Bol and van de Werfhorst, 2013; Kerckhoff, 2001). They are also less directly applicable to influencers whose authority is based primarily on lifestyle aspiration, motivational self-branding, or consumer identity rather than on guidance around gatekeeping educational decisions. Risk translation and compressed meritocracy should therefore be understood as concepts that may be extended to other settings only with careful attention to institutional context. Their broader relevance requires comparative evidence before they can be generalized beyond China's *Gaokao* and postgraduate-admission ecology.

To analyze the Zhang Xuefeng phenomenon as a form of risk translation within a compressed meritocracy, the following sections develop two complementary lines of argument. The first explains the sociological mechanisms that made Zhang's message socially intelligible. The second explains the social-psychological mechanisms that made his advice persuasive in individual and family decision-making.

## 3 Sociological mechanisms: when educational choice becomes risk management

If compressed meritocracy describes the structure within which families make educational decisions, credential inflation helps explain why Zhang's advice appeared sociologically intelligible (Lin et al., 2024). As higher education expands, degrees may continue to improve life chances, but their relative scarcity declines. Credentials that once distinguished graduates can become baseline requirements, pushing students to seek further differentiation through institutional rank, field of study, postgraduate qualifications, internships, professional certificates, city location, and social networks. Collins (1979) account of the credential society is useful here because it shows that credentials function not only as indicators of skill but also as currencies in competition for organizational positions. Research on China's educational expansion supports this interpretation. The massification of higher education has widened access, but it has also complicated graduate employment and weakened the mobility returns of educational attainment for some groups (Mok and Wu, 2016). Family background continues to shape educational enrollment and transitions, with rural students facing greater disadvantages at later stages of the educational process (Wu, 2010). Hukou-linked *Gaokao* policies further illustrate this inequality. As students are often required or strongly encouraged to take the *Gaokao* in the locality of their household registration, these policies can disadvantage children of rural-to-urban migrant families who study outside their place of household registration (Li and Zhang, 2023). Educational expansion therefore allows more students to enter the competition, but it does not ensure that all students can convert participation into equal educational or labor-market returns.

This problem of conversion brings information inequality to the center of the Zhang Xuefeng phenomenon. Formal education systems often assume that students choose majors based on interests, aptitudes, and counseling. In practice, however, the long-term value of a major is unevenly distributed as social knowledge. Families with professional networks may understand which credentials are flexible, which universities carry regional value, and which fields contain hidden barriers. Families without such networks often rely on rankings, hearsay, digital platforms, and visible success stories. Zhang's public role can be interpreted as an attempt to make informal institutional knowledge more accessible. He spoke as if he were revealing the informal rules and hidden constraints of the education-to-work transition: which programs convert poorly into employment, which credentials are overvalued, and which choices offer occupational closure for students with little room for error.

Zhang's advice was not always accurate or uncontroversial, and it should not be treated as neutral expertise simply because many families found it useful. Its appeal nevertheless revealed an institutional deficit. When official advising is perceived as generic, overly promotional, or disconnected from labor-market realities, families often turn to alternative interpreters. Granovetter (1973) theory of weak ties helps explain why occupational information frequently circulates beyond the immediate family and through broader social networks. In the digital platform era, influencers can reproduce this weak-tie function at scale by giving audiences access to information they may otherwise lack. Zhang's blunt, anti-pretentious style made him appear closer to families with fewer socioeconomic resources than to institutional elites. His influence, therefore, rested not only on the information he provided but also on his performance of class proximity and practical solidarity.

A balanced account should also recognize the counter-narratives around Zhang. His comments about journalism majors drew criticism from academics who argued that his employability-first framing reduced a broad field of study to a narrow job-matching calculation, while Zhang defended his position as practical advice for students from families with fewer socioeconomic resources (Liang, 2023). His later remarks associating liberal arts majors with service work triggered a major online controversy and were criticized as derogatory and excessively utilitarian (Quan, 2023). These controversies matter analytically because they suggest that risk translation can clarify unequal realities while also oversimplifying disciplines, stigmatizing fields of study, or turning legitimate employment anxiety into deterministic advice. The point is not that Zhang was always right, but that even contested advice can become influential when it speaks to anxieties that formal institutions have not sufficiently addressed.

## 4 Social-psychological mechanisms: why blunt advice may have felt like care

Sociological conditions created the demand for Zhang's advice, but social psychology helps explain why his particular style may have been persuasive. To avoid treating multiple theories as a loose list of compatible explanations, the psychological argument can be organized as an integrated sequence. First, uncertainty created demand for structure; second, perceived class proximity and bluntness made Zhang appear credible; third, loss-focused framing made advice emotionally urgent; fourth, social comparison amplified families' sense that choices were relational and consequential; and fifth, repeated social media exposure created parasocial familiarity. Table 1 summarizes how each mechanism contributes to the overall architecture of the argument.

**Table 1:** Integrated social-psychological architecture of the Zhang Xuefeng phenomenon.

Aspect of the phenomenon	Mechanism explained	Main theoretical resources
Demand for decisive rules	Ambiguity and high-stakes decisions increase the appeal of clear, actionable guidance.	Need-for-closure theory; uncertainty-identity theory
Perceived credibility	Blunt speech and class-coded solidarity make the adviser appear to represent the interests of families with fewer socioeconomic resources.	Source credibility; epistemic authority; class proximity
Emotional urgency	Potential loss, unemployment, and downward mobility are weighted more heavily than open-ended exploration.	Prospect theory; scarcity theory
Social amplification	Families interpret scores, majors, and universities relationally through comparison with other families and cohorts.	Social comparison theory; platform visibility
Perceived care and attachment	Repeated exposure turns a public adviser into a familiar surrogate counselor who provides structure and relatedness.	Parasocial interaction; self-determination theory

Need-for-closure theory suggests that ambiguity can generate a desire for firm answers, especially when decisions appear consequential or difficult to reverse (Kruglanski and Webster, 1996). Uncertainty-identity theory similarly argues that individuals facing uncertainty may be drawn to clear and authoritative positions that reduce confusion (Hogg, 2007). Zhang's lists, rankings, warnings, and direct prescriptions reduced the cognitive burden of educational decision-making. Their decisiveness may have made them psychologically useful, even when their certainty was open to debate. In addition, trust in Zhang likely depended on perceived alignment. Source credibility is not only a matter of expertise. It also involves judgments about whose interests the speaker appears to represent. Zhang's public persona was not that of a distant policy expert. He mocked empty prestige, named uncomfortable realities of the labor market, and used a conversational style closer to everyday speech than to official counseling discourse. Families may have trusted him not only because he seemed to understand the system, but also because he appeared willing to say what polite institutions avoided. In this sense, he became an epistemic authority by confirming the audience's suspicion that idealized educational language could conceal unequal risks.

Social comparison further strengthened this authority. Social comparison theory suggests that people evaluate themselves in relation to others when objective standards are insufficient (Festinger, 1954). Chinese education is filled with scores, ranks, and cutoffs, but their meanings remain relational. A score matters because of how it positions a student within a province, cohort, institution, or labor market. Online platforms intensify such comparisons by making other families' strategies highly visible. Zhang organized this noisy comparison environment into narratives of ladders, traps, winners, and preventable mistakes. In doing so, he organized anxiety into a narrative structure centered on avoidable loss. As noted earlier, prospect theory holds that potential losses often weigh more heavily than comparable gains (Kahneman and Tversky, 1979, 2013). Research on scarcity further shows that constrained resources direct attention toward urgent trade-offs and immediate threats (Shah et al., 2012). For families with limited buffers, the major choice is not a neutral process of exploration. It is a decision about whether years of parental investment can be protected from unemployment, underemployment, or prolonged dependence. Zhang's employability-first advice, therefore, may have felt harsh and caring at the same time: harsh because it narrowed the range of imaginable futures, but caring because it promised protection against irreversible loss.

Social media may also have given Zhang a form of parasocial authority. Horton and Wohl (1956) described parasocial interaction as the illusion of intimacy that audiences develop with media figures. Through repeated exposure on digital platforms, Zhang appeared not only as an adviser but also as a familiar voice in family decision-making. For students and parents who lacked access to individualized counseling, he could serve as a surrogate counselor or an elder-brother figure. His influence suggests that educational authority can be produced not only through institutional position or professional certification, but also through affective familiarity and perceived solidarity. This does not mean that educators should imitate fear-driven pragmatism. Self-determination theory offers a more constructive interpretation. Students need autonomy, competence, and relatedness (Deci and Ryan, 2000), but autonomy without reliable information can feel more like abandonment than freedom. Zhang supplied structure, though often in an occupationally narrow and emotionally pressurized form. The educational challenge is therefore to provide guidance that is equally concrete while also preserving students' dignity, exploration, and developmental breadth.

## 5 From public grief to democratic guidance

Zhang's death intensified the phenomenon by linking educational anxiety to bodily exhaustion. Sociologically, its symbolism was difficult to ignore: a man who taught families how to navigate an overcompetitive education system died young in a society already concerned about overwork, exhaustion, and pressure on young people. Public grief may therefore be interpreted as more than celebrity mourning. It appeared to function as a moral event in which one individual's life condensed a broader social contradiction. People might have mourned Zhang as an interpreter of the system, while also mourning the conditions that had made such an interpreter necessary. Merton (1938) strain theory helps clarify this contradiction. When societies strongly emphasize culturally valued goals, such as upward mobility, but distribute legitimate means unevenly, strain is displaced into families, schools, and bodies. Zhang's advice both reinforced and exposed this strain. He accepted the rules of competition by urging students and families to adapt strategically, while also making the harshness of those rules publicly speakable. This ambivalence can also be understood through system justification theory, which suggests that people may seek stability within existing systems even while recognizing that those systems produce unequal burdens (Jost and Banaji, 1994).

For educators, the lesson is neither to reject pragmatism nor to surrender education entirely to market prosperity. The more important task is to build democratic guidance for a risk society. Democratic guidance in this context refers to the guidance that makes high-quality educational and labor-market information more equally accessible, interpretable, and usable across socioeconomic groups. Such guidance should provide transparent information about employment outcomes, graduate pathways, professional licensing, internships, regional demand, and the hidden forms of capital required by different fields. It should also be equity-sensitive, recognizing that the same major can carry different implications for students with different economic, cultural, and social resources. In addition, educational guidance should cultivate decision literacy, helping students evaluate uncertainty, opportunity costs, transferable skills, and fallback options. Career decision-making research shows that students often need structured support to identify information gaps, evaluate inconsistent information, and move from indecision to reasoned choice (Gati and Levin, 2014). A social-justice approach to career guidance further requires counselors to address unequal resources and opportunity structures rather than treating career choice as an isolated individual preference (Hooley and Sultana, 2016). Guidance should also treat mental health as part of educational quality, especially given evidence of substantial depressive symptoms among Chinese children and adolescents (Zhou et al., 2024). Above all, effective guidance should combine structure with autonomy: enough realism to prevent abandonment, and enough imagination to prevent fatalism (Liu H. J. et al., 2026).

These principles can be operationalized in concrete ways. Labor-market transparency, for example, could involve school-level or provincial dashboards that report graduate employment rates, typical salary ranges, postgraduate entry routes, civil service eligibility, licensing requirements, and regional demand by major, while also explaining the uncertainty and limitations of such data. Equity-sensitive advising could involve counseling interviews that ask not only “What are you interested in?” but also “What risks can your family reasonably absorb?,” “What support networks do you have?,” and “What alternative routes remain open if the first plan fails?” Decision literacy could be taught through scenario-planning exercises in which students compare several major-and-career pathways, identify reversible and irreversible choices, map transferable skills, and discuss how to balance employability with personal meaning. Mental-health integration could mean that high-stakes advising sessions include referral routes for anxiety, family conflict, and burnout, rather than treating distress as a private weakness. These examples illustrate that democratic guidance is not abstract encouragement; it is a practical institutional capacity to make risk visible, discussable, and less unequally distributed.

The Zhang Xuefeng phenomenon suggests that families did not turn to blunt online guidance because they had stopped valuing education. They turned to it because education had become both indispensable and unpredictable. Zhang translated risk into rules, giving anxious families a sense of control. If educators wish to reclaim trust, they must become better translators than influencers. This means more evidence-based than rumored, more humane than fearful, and more social awareness than obfuscated aspiration. At the same time, the explanation offered here remains bounded by the limits of a single case and secondary evidence. Comparative research on other edu-influencers, career coaches, and exam-advice markets inside and outside China is needed to determine when risk translation becomes democratic guidance, when it becomes commercialized fear mongering, and when it becomes both at once. The future of educational guidance does not lie in choosing between passion and survival. It lies in building institutions in which students from less advantaged backgrounds can pursue meaningful lives without being required to gamble their families' futures.

## Data Availability

The original contributions presented in the study are included in the article/supplementary material, further inquiries can be directed to the corresponding author.
